# Supplementary material for: Are carbon emissions trading and green financial instruments synergistic? -Comprehensive quantitative research based on content analysis
Source: PLoS One. 2024 Mar 7;19(3):e0298601. doi: 10.1371/journal.pone.0298601 (PMC10919601; doi:10.1371/journal.pone.0298601)
Supplement: S1 Data — (ZIP) [file pone.0298601.s001.zip › Data/地级市Z_F工作报告.zip!/╜¡╬≈/╛┼╜¡╩╨/└┤╫╘╣1⁄2╓┌║┼í╛┬φ┐╦╩2╛▌═°í┐_2009.docx]

一、2008年工作简要回顾。

2008年，在省委、省政府和市委的领导下，市政府深入贯彻落实科学发展观，紧紧依靠全市人民，开拓进取，扎实工作，完成了市十三届人大三次会议提出的任务。全市生产总值700.59亿元，比上年增长12.5%；财政总收入66.17亿元，增长15.0%，其中地方一般预算收入38.42亿元，增长19.8%；城镇固定资产投资439.76亿元，增长31.7%；社会消费品零售总额201.94亿元，增长23.3%；居民消费价格总水平上升5.7%，低于全国全省涨幅；城镇居民人均可支配收入12889元，增长14.3%；农民人均纯收入4417元，增长12.2%；万元生产总值能耗下降4%，化学需氧量和二氧化硫排放量控制在省下达指标之内，分别下降4.7%和5.95%；人口自然增长率控制在7.67‰。一年来，主要抓了以下几个方面的工作：

（一）突出第一要务，经济实现平稳发展；

（二）深化改革开放，经济社会发展活力和动力增强；

（三）加强基础设施建设，发展环境进一步改善；

（四）沿江开发取得新进展，临港经济呈现良好势头；

（五）推进鄱阳湖生态经济区建设，生态建养力度加大；

（六）社会事业协调发展，人民群众得到更多实惠；

（七）坚持依法行政，政府自身建设不断加强。

二、2009年工作安排。

（一）做强园区，推动工业经济跨上新台阶

工业大则总量大，工业强则实力强。在把握大局上突出工业这个核心，在资金投入上突出园区这个平台，在服务发展上突出项目这个重点，营造不遗余力建园区、倾心竭力上项目、举全市之力主攻工业的浓厚氛围，形成大手笔建大平台、大气魄上大项目、大作为成大产业的工业发展新格局。实现全年工业投资450亿元，增长50%，规模以上工业增加值300亿元以上，主营业务收入突破1000亿元。

创新园区建设，做实工业平台。把开发区、园区作为发展工业的主战场，创新体制，夯实基础，做优环境，提升人气，使之成为发展自主权最大、人员配备最强、办事效率最高、设施条件最优的工业平台。

推进重大项目，做强工业支柱。瞄准重大项目抓落户，围绕龙头企业搞扩改，改变工业“旗舰”不大、“航母”不多的现状，形成大项目带动、大企业支撑的良好势头。加强项目审核和调度，坚决防止以小报大、以弱充强、以假乱真；坚决防止抓而不实、推而不动、建而不快；认准大项目、抓住大项目、建成大项目。

扶持中小企业，做大工业集群。针对金融危机给企业生产经营造成的冲击和影响，特别要帮助危机影响大、竞争实力弱的中小企业度过难关，从政策、资金、市场等方面加大扶持、强化服务。

加快国企改制，做活工业存量。

（二）振兴城区，推动城市建设走进新时代

发挥城市化对新型工业化和农业现代化的撬动作用，以拉大框架、完善功能、提升品位、繁荣经济为主线，规划坚持高品位、建设突出快速度、质量追求高标准，推动城建重心走进“八里湖时代”。

科学规划城市。按照2015年之前实现中心城区100平方公里、100万人口的目标，遵循主攻新城区、挺进八里湖、完善老城区、统筹庐山区、挖掘古浔阳、拉大中心城区框架的思路，年内完成城市总体规划修编，配套完善土地、水系、路网等各类专项规划，重点做好八里湖、甘棠湖、南门湖、十里河、龙开河水系规划，使之自然涵养、畅通活化、旱涝可调。按照“行政文化中心、最佳人居新区、休闲观光乐园”的定位，高起点、高品位编制八里湖组团详规。强化规划的刚性约束力，建立健全城乡规划监管机制。

科学建设城市。坚持城市建设项目化，把城市“西拓、东延、南移、中疏”落实到具体项目上。每年新增投入10-20亿元，突出建设八里湖组团，协调推进其它组团，逐步把九江建设成为一个山水辉映、环境优美、功能完善、宜居宜业的沿江港口城市。

科学经营城市。加快城建靠资金，资金来源靠经营，经营重点在土地。十分珍惜土地资源，加大规费征缴力度，千方百计为做大城市筹集资金。一是加强土地运作；二是加强规费收缴；三是加强资产融资。

科学管理城市。管理见水平，管理出品位，品位显魅力。一是实施“创建工程”，开展文明城市、卫生城市、园林城市、国家级历史文化名城创建活动，以净化、美化、亮化为重点，加强“三区”结合部、城区与园区结合部、城乡结合部建设和管理；加强城市古建筑保护和修复，挖掘古浔阳文化内涵，对自然景观注入文化意蕴。二是实施“绿化工程”，全面推进中心城区扩地增绿、拆墙透绿、立体披绿，最大限度增加绿化总量，打造绿化精品，逐步形成一路一特色、一街一景致。三是实施“畅通工程”，疏通交通瓶颈，构建街区微循环，增加停车场所，缓解干道压力，强化交通管制，倡导文明出行。理顺体制，完善线网，更新车辆，提升公交档次。四是实施“平安工程”。

科学繁荣城市。一是提升传统服务业，完善商业网点布局，加快形成一批专业特色街、品牌旗舰店和大型卖场，打造中心商贸区；支持京九农副产品批发市场改造升级，推进华东装饰材料市场、南方粮食交易市场、九江国际汽车城等重点市场加快发展。二是发展现代服务业。三是做大城市物流业，加快港口和电子物流信息平台建设，发展水陆联运，支持九江机场扩大空运，积极开辟物流新通道。四是改善城市住房条件，合理确定经济适用房、廉租房和商品房比例，正确引导房价和市场预期，促进房地产市场健康发展。

（三）夯实基础，推动“三农”工作迈出新步伐

推进农村制度改革。实行最严格的耕地保护和节约用地制度；允许土地承包经营权依法、自愿、有序流转，推动适度规模经营；深化林权制度配套改革，规范林业产权交易，拓展林权质押贷款和林业保险业务；推进国有农场税费改革和综合配套改革；落实强农惠农政策，将种粮、良种、生猪养殖、农机具购置和农用成品油、化肥价格补贴等发放到位；完善补偿和保障机制，确保失地农民生活水平有提高，长远生计有保障。

强化农村发展基础。实施“沃土工程”、“造地增粮富民工程”、“新增百亿斤优质商品粮生产能力工程”，改造中低产田8万亩。

壮大农业产业规模。高度重视粮食安全，粮食种植面积稳定在360万亩以上，总产量27亿斤以上。大力培植优势产业，发展特种水产和水产品加工，抓好标准化农产品生产示范基地和鄱阳湖现代农业产业带建设，加强鄱阳湖渔业资源深度开发，巩固发展九江水梨。推进农业项目建设，每个县(市、区)力争引进3000万元以上农业项目2-3个，亿元以上项目1-2个，市直农口各单位引进3000万元以上农业项目1-2个；积极申报一批农村经济社会发展项目，力争更多地争取国家和省级农业项目资金。

完善农业服务体系。加大农业科技投入，抓好“粮食丰产科技工程”等农业科技计划项目的实施。

（四）扩大开放，推动项目投资取得新突破

坚持开放引领，突出投资拉动，以开放引项目，以项目扩投入，以投入保增长。

加大招商引资力度上项目。抓住全球投资看好中国、国内客商看好中部的有利时机，创新开放举措，加快产业和市场对接，针对性地组织招商活动，努力提高招商实效。

　　加大对接跟踪力度争项目。用足用好国家扩大投资、拉动内需的各项政策，研究动向，超前论证，科学包装，频密对接，全程跟踪，力争全市争资份额超过全省平均水平。

加大环境整治力度落项目。

（五）激发活力，推动县域经济实现新跨越

按照三年实现财政收入县县过5亿，一批县区过10亿、15亿的目标，放开放活，强化激励，八仙过海，各显神通，促进县域经济做大做强。

给足发展权、铺好就业路、掀起创业潮、打好擂台赛。

（六）扬优成势，推动特色板块形成新亮点

扬沿江水运之优，成临港产业集聚之势。

扬庐山品牌之优，成全市旅游联动之势。

扬湖区生态之优，成绿色经济衍生之势。

扬共青独特之优，成青年创业蓬勃之势。

（七）改善民生，推动社会事业再上新水平

按照人民政府为人民的要求，结合九江实际，尽最大的努力实施民生工程，尽最大的努力办好惠民实事。重点围绕八个方面，办好89件实事。

一是努力扩大就业。着力办好8件实事，主要是：以创业带就业，新增小额担保贷款1.87亿元，支持个人创业和劳动密集型小企业发展。加强就业培训，培训6.6万农村劳动力；免费为5.1万下岗失业人员、城镇登记失业人员、未就业高校毕业生、城镇复员退伍军人提供技能和创业培训。扶持困难群体就业，确保有就业愿望和能力的零就业家庭至少有1人就业，为残疾人购买390个公益性岗位。将符合条件的被征地农民、农林水失业人员、未安置的城镇退役军人等统一纳入失业登记范围，享受就业扶持政策。力争新增城镇就业5.21万人，其中 “4050”人员5400人；新增转移农村劳动力4.3万人，其中省内转移2.41万人。

二是进一步提高社会救助能力。着力办好14件实事，主要是：提高城乡低保对象补助标准，月人均补差分别达到155元、65元。提高农村五保户供养标准，年人均提高60元。提高上世纪六十年代精简下放老职工救济金水平，城乡月人均分别达到200元、160元。提高城镇“三无”特困群众供养标准，月人均提高到300元。提高义务兵优待金标准和退役士兵自谋职业补助水平，增加企业军转干部和部分退役军人生活补助。完善临时救助制度，配套资金156万元，对因突发性事件造成生活困难的群众实行临时救助。建立因自然灾害死亡人员抚慰金制度，按每人5000元标准抚慰家属。对困难群众实施法律援助，完成法律援助案件1200件以上。对符合条件的计划生育扶助对象，每年发放720元奖励扶持金，让计划生育家庭真正得到更多实惠。新建市儿童福利院和2所县级综合福利院。为1万名残疾人提供康复服务，为900名贫困白内障患者实施复明手术，为2400名贫困精神病患者实施医疗救助，为100名贫困残疾人配发辅助器具，让残疾人感受到更多的关爱和温暖。

三是不断提升社会保障水平。着力办好9件实事，主要是：扩大养老保险覆盖面，城镇职工参保人数达61.99万人。提高企业退休人员养老金水平，人均增加140元。推进被征地农民养老保险试点，新增参保1.5万人。扩大城镇职工工伤保险覆盖范围，参保人数达25.35万人。提高失业保险统筹水平，参保人数达34万人。调整园区职工社保缴费基数和计算标准，接续返乡农民工社会保险关系。改扩建40所农村敬老院，建设一批县区老年公寓。对离任老村支书、村主任给予生活补贴，对敬老院、福利院、光荣院“三院”中符合条件的工作人员以及居委会工作人员给予工作补贴，让长期工作在最基层的同志生活上得到应有照顾。

四是大力加强医疗保障和食品药品安全。着力办好15件实事，主要是：扩大城镇职工基本医疗保险覆盖范围，参保职工达51.2万人。完善城镇居民基本医疗保险制度，将大学生全部纳入城镇居民基本医疗保险范围。提高新型农村合作医疗保障水平，参合农民达到315万人，统筹基金使用率达85%。全市配套救助资金1477万元，提高大病医疗救助水平。免费向符合条件的城市居民发放公共卫生服务卡490万元。完善城乡卫生服务体系，建立100家农村示范药店，推进344个村卫生室（所）、72个社区卫生服务机构、13所乡镇中心卫生院、2所县级综合医院、1所县级中医院达到建设标准。新招聘农村卫生专业技术人员1000名，免费培训农村卫生技术人员2000名。按每人每年1000元标准对符合条件的村卫生室执业医生给予补助。乡镇卫生院工作人员基本工资全部由差额转为全额保障。

五是继续大力推行济困助学。着力办好10件实事，主要是：增加城乡义务教育发展经费，继续实施“两免一补三资助”。为义务教育阶段59.5万名学生免除学杂费、免费提供教科书，对3.67万名义务教育阶段家庭经济困难学生补助寄宿生活费，资助家庭经济困难的高中生1万人以上、中专生2.8万人以上、大学新生2700人以上。免费录取540名家庭经济困难学生入读技工学校。新招聘1000名农村教师。改造农村中小学校舍8万平方米。对边远地区教师发放特殊津贴。今年所有县（市、区）公务员津贴补贴和义务教育教师绩效工资要全部发放到位。

六是加大保障性住房建设力度。着力办好5件实事，主要是：扩大廉租住房保障，力争新建廉租住房5000套，租赁补贴1.2万户。继续推进经济适用住房建设，受益家庭达到1570户。启动实施中心城区20万平方米棚户区（城市低洼危旧房住宅区）改造。建立农村困难群众危房救助制度，支持帮助732户农村困难群众改造危旧住房，逐步改善困难群众的住房条件。

七是扎实推进扶贫开发。着力办好3件实事，主要是：继续实施移民搬迁扶贫，力争完成深山区、库区、地质灾害频发区贫困群众5600人移民搬迁。大力实施“整村推进计划”，重点抓好357个重点村扶贫开发。推进农业产业化扶贫，扶持产业化扶贫龙头企业发展和产业化基地建设，扩大贫困村互助资金试点范围，帮助贫困农户通过发展生产增加收入、改善生活。

八是切实改善人民群众生产生活条件。着力办好25件实事，主要是：改善群众出行条件，开通城区至城西港区等郊区公交线路。加大改水力度，新增解决6万农村群众安全饮水问题。推行清洁能源，增加农村沼气用户2.28万户。加强农村环卫设施建设，新建农村家庭生活垃圾处理场692个。实施“万村千乡市场工程”，新建标准化农家店100家以上。实施农村光缆进村工程，使行政村宽带网络通达率达到90％以上。实施广播电视村村通工程，帮助654个20户以上自然村开通卫星电视。建设乡镇文化站39个，建成农家书屋219个。开展农民体育健身场所工程建设试点，新建农民体育健身场所51个。进一步优化城区人居环境，完成城区自来水一户一表改造1.5万户以上，启动江州菜市场等一批农贸市场升级改造。进一步完善公共服务体系，设立就业服务热线和网络平台，整合阳光政务热线，畅通民声通道。在端午节、儿童节、中秋节、重阳节，向全市人民免费开放观光游览景区景点。

与此同时，全面发展社会事业。一是优先发展教育。二是推动科技进步。三是加强卫生建设。四是大力发展文体事业。五是做好人口人才工作。

三、加强政府自身建设。

2009年将是我国改革开放以来，尤其是新世纪以来经济最困难、形势最复杂的一年，我们既要有充分的估计，又要有坚定的信心。困难面前勇者胜。我们一定要把思想行动统一到胡锦涛总书记视察江西时提出的“三保一弘扬”要求上来，统一到省委省政府准确分析新形势、积极应对新挑战所作出的一系列重大部署上来，统一到市委确定的目标任务和战略抉择上来，以“对党负责、为民服务、高效务实、清正廉洁”的政府团队精神，全力以赴，攻坚克难，把强工兴城的决策落实好，把奋起赶超的目标实现好。

（一）提高操作能力保增长。

（二）提高统筹能力保民生。

（三）提高驾驭能力保稳定。

（四）提高拒腐能力保清廉。

各位代表！三年翻番，只争朝夕；五年进位，时不我待。让我们在省委、省政府和市委的正确领导下，在市人大、市政协的监督支持下，坚定信心，应对挑战，齐心协力，奋起赶超，全面完成市委确定的各项目标任务,以优异的成绩向新中国60华诞献礼！

2009年政府工作报告（全文）

各位代表：

现在，我代表市人民政府，向大会报告工作，请予审议，并请各位市政协委员和列席会议的同志提出意见。

一、2008年工作简要回顾

2008年，在省委、省政府和市委的领导下，市政府深入贯彻落实科学发展观，紧紧依靠全市人民，开拓进取，扎实工作，完成了市十三届人大三次会议提出的任务。全市生产总值700.59亿元，比上年增长12.5%；财政总收入66.17亿元，增长15.0%，其中地方一般预算收入38.42亿元，增长19.8%；城镇固定资产投资439.76亿元，增长31.7%；社会消费品零售总额201.94亿元，增长23.3%；居民消费价格总水平上升5.7%，低于全国全省涨幅；城镇居民人均可支配收入12889元，增长14.3%；农民人均纯收入4417元，增长12.2%；万元生产总值能耗下降4%，化学需氧量和二氧化硫排放量控制在省下达指标之内，分别下降4.7%和5.95%；人口自然增长率控制在7.67‰。一年来，主要抓了以下几个方面的工作：

（一）突出第一要务，经济实现平稳发展

全市规模以上工业增加值228.05亿元，增长20.3%；规模以上工业企业主营业务收入833.06亿元，增长29.8％。新增开工、投产工业项目123个，完成投资291.17亿元，增长61.7%。九钢120万吨高线技改、巨石集团30万吨玻纤、龙达30万吨化纤、赛得利二期、旭阳雷迪多晶硅、诺贝尔陶瓷等一批重大项目开工建设，钢铁、化纤、船舶、建材等产业集聚势头良好；工业园区集约发展水平提升，全市工业园区主营业务收入突破500亿元，九江开发区主营业务收入超过100亿元，万福工业园成为我省节能灯产业基地。

粮食总产144.98万吨，实现连续五年丰收；水产品总产35.71万吨，其中特种水产10.5万吨；棉花种植73.7万亩，总产8.21万吨；油料产量13.54万吨；生猪养殖向专业化、规模化发展，出栏171.64万头。农业产业化扎实推进，培育国家级龙头企业1家、省级龙头企业18家、省级以上农业标准化示范基地11个，创绿色、有机、无公害食品品牌43个，新增专业合作社308家，扩种九江水梨6万亩。新农村建设继续走在全省先进行列，投入2.1亿元，建设1473个试点村，农民生产生活条件得到改善，农村面貌有了新的变化。

克服金融危机影响，全市金融机构贷款余额338.79亿元，比年初增加76.76亿元；九江银行与兴业银行开展战略合作，迈出跨地区发展步伐，浦发银行、招商银行等9家金融机构落户九江；江洲联合造船在香港联交所借壳上市；积极开展政银企合作，签订贷款合同63.69亿元。沃尔玛等知名零售企业入驻九江，华东市场三期竣工开业。启动庐山园门下迁和庐山新城建设，庐山西海风景区总规通过省政府审批；庐山机场开通北京、上海、广州、厦门四条航线，旅客由上年的7000人次上升至4万余人次；举办金秋经贸文化旅游节暨第三届国际龙狮精英赛和真如禅寺1200周年庆典，被评为“2008中国十大特色休闲城市”，实现旅游总收入96.7亿元，同比增长8.8%。

（二）深化改革开放，经济社会发展活力和动力增强

积极推进国企改革，一批市属企业改制基本完成；国有农场税费改革和林权制度配套改革不断深化；扎实推进投融资体制改革，组建了沿江开发建设投资公司、城投公司和市投资担保公司；继续深化事业单位岗位设置管理改革；完善行政服务中心建设，组建了公共资源交易中心；乡镇机构改革全面完成，所有乡镇推行"乡财县代管"。

有效开展对接招商活动，全市实际利用外资4.36亿美元，引进省外5000万元以上的工业项目资金113.08亿元。外贸进出口总额4.79亿美元，同比增长32.6%。对外工程承包额1.4亿美元，出境劳务由上年850人增加到2558人。新增国际友好城市3对，荣获国际友好城市交流合作奖。民营经济加快发展，新增民营企业2204户，税收总额达到30.58亿元，占财政总收入的46.2%。

（三）加强基础设施建设，发展环境进一步改善

铜九铁路全线通车，武吉高速基本建成，昌九城际铁路、九瑞高速、彭湖高速顺利开工，西气东输过江盾构工程加速推进。继续狠抓“三年打胜公路建设翻身仗”，沙阎公路、机场路等改造完成，硬化农村公路1773公里，新增310个行政村通水泥路，完成农村改渡建桥10座，农村公路通畅率86%，通达率90%以上，6个县（市、区）基本实现村村通；48座病险水库可在主汛期前完成除险加固主体工程。矶山湖、长岭风电并网运行，彭泽核电征地拆迁和移民安置工程、九江电厂四期前期工作进展顺利。城市建设稳步推进，城镇化率达到43.05％。续建14条城区道路，完成100条背街小巷整治。长途客运中心、公交总站、两湖活化引水工程顺利推进，水、电、气、公交等设施不断完善，八里湖污水处理厂一期工程投入试运行，市区生活污水集中处理率达到60%以上。南湖公园入选全国第二批国家重点公园，武宁县城被评为国家级园林县城。

（四）沿江开发取得新进展，临港经济呈现良好势头

编制完成《九江市港口总体规划》、《城西港区控制性详细规划》。投入20.4亿元，加快建设沿江道路、供排水、电力、防洪等基础设施。城西港区30万标箱集装箱码头开港运营，港区物流园区一期工程完工，疏港大道建成通车，安置小区基本建成，通关中心开工建设，口岸扩大开放纳入国家规划，九江港获准对台湾港口直航。沿江开发对投资者的吸引力进一步增强，金砂湾、码头、城东工业集中区、九江县沿江产业集群区等板块承载力进一步提升，工业集聚势头良好，一批冶金、建材、船舶、化工等重大工业项目相继开工投产。

（五）推进鄱阳湖生态经济区建设，生态建养力度加大

参与编制鄱阳湖生态经济区总规和18个专项规划，完成九江鄱阳湖滨湖保护区的功能区划；鄱阳湖生态枢纽工程等一批项目列入省规划。积极推进重点领域和重点行业的节能减排，14户重点企业单位能耗持续下降，全市重点污染源在线监控系统和市区大气、水质、噪声自动监测监控系统建成；县级污水处理厂建设顺利推进。加强农村面源污染控制，探索开展农村生活垃圾无害化处理；实施修河环保行动，关闭一批沿河污染企业，修河水质进一步改善。大力支持共青城跨越式发展，积极申报共青城青年创业基地、台商创业园和农业示范基地。

（六）社会事业协调发展，人民群众得到更多实惠

市县财政配套4.14亿元，全面完成省政府下达的民生工程8大类81项指标。新增城镇就业6.8万人、“4050“人员就业7500人；发放小额贷款1.61亿元，比上年增加5554万元，扶持4003名下岗失业人员自主创业，带动10474人就业；征缴养老保险金8.8亿元，增长近2亿元，备付能力进一步提升；提高城乡低保补助标准，月人均补差分别增加35元、17.9元，农村低保对象由10万人增加到16.4万人；扩大医疗救助范围，取消了病种限制，提高了救助标准；农村五保集中供养率达84%，农民参加合作医疗人数314.5万人；补助义务教育阶段贫困寄宿生生活费3237万元，资助贫困大学生9261人。启动20万平方米经济适用住房建设，惠及3000户城市中低收入家庭，推进廉租房和经 济适用房建设向县级拓展。去年政府提出的十件实事逐项推进，完成良好。

全市获省级科技进步奖4项，行业科技进步奖2项；小学适龄儿童入学率达99.99％，初中少年入学率达98％，万人高中学生比达347，居全省首位，新招聘教师1200名，九江学院、九江职大、九江职业技术学院办学水平不断提高；三所医院通过省三级甲等医院评审，都昌县代表全省通过国家血吸虫病疫情控制考核。计划生育基础服务能力进一步增强，人口出生性别比失衡得到有效控制；竞技体育在奥运会上取得历史性突破；公民道德建设得到加强，涌现出见义勇为、舍生取义的英雄人物许俊谷景；扶贫、慈善工作走在全省前列，募集超亿元救灾款物支援四川抗震救灾。审计、统计、质监、药监、民族宗教、国家安全工作进一步加强，档案、供销、农垦、气象、水文、地震、烟草、信息化、地方志等工作取得新成效，文学艺术、新闻出版、广播电视事业健康发展，妇女、儿童、老年人、残疾人权益得到保护。

认真落实安全生产责任制，全市没有发生重特大安全事故；深入开展社会治安综合治理，社会治安形势明显好转；国防教育和民兵预备役工作得到加强；加大力度调处信访突出问题，赴京赴省集体访、异常访大幅下降，保持了社会和谐稳定。

（七）坚持依法行政，政府自身建设不断加强

加强民主法制建设，自觉接受市人大的依法监督和市政协的民主监督，认真向人大报告工作，向政协通报工作，真诚接受各民主党派、工商联和人民团体参政议政。办理市人大代表建议186件，市政协提案400件，满意率有明显提高。扎实开展民主评议政风行风和机关效能建设提高年活动；建立开通了市政府信息公开网上平台，完善新闻发言人制度。加强勤政廉政建设，行政权力运行监控机制进一步完善，惩治预防体系进一步健全。

各位代表，纵观去年的政府工作，改革深化、发展平稳、民生改善、社会稳定。这一年，战胜了历史罕见的冰雪灾害，积极应对金融危机带来的不利影响，实现了经济平稳发展；推进了一批重大项目，发展后劲进一步增强；实施了民生工程，群众生产生活条件得到改善。这些成绩的取得，是省委、省政府和市委正确领导的结果，是市人大、市政协监督支持的结果，是全市人民和社会各界人士团结奋斗的结果。在此，我代表市人民政府，向辛勤劳动在各个领域的全市人民，向大力支持政府工作的各位人大代表、政协委员，向离退休老同志，向各民主党派、工商联、各人民团体和社会各界人士，向驻浔部队和武警官兵，向中央和省驻浔各单位，向所有关心、支持、参与九江建设和发展的海内外各界朋友，表示衷心的感谢并致以崇高的敬意！

在肯定成绩的同时，我们也清醒地看到，在全省竞争发展的格局中，九江的位次已经后移，多项指标排位后列；园区、城区发展相对滞缓；产业层次偏低，市本级支柱财源单一，县域经济底子较弱；国企改制、农民增收、就业和再就业等难点问题仍待攻坚；政府及其部门转变职能、提升效能亟待进一步加强。对此，我们将直面困难，针对问题，深入研究，努力解决。

二、2009年工作安排

2009年是新中国60华诞的喜庆之年，是我市奋起赶超、争得应有地位的起步之年。尽管当前发展受到国际金融危机的影响，但是国家针对性地出台了一系列保增长、促发展的重大举措，我们应对挑战、奋起赶超有良好的机遇；尽管在激烈的区域竞争中，我们的位次后移，但是市委审时度势，作出了新的战略抉择，我们直面竞争、奋起赶超有充足的信心；尽管我们争得应有地位将会遇到巨大的压力和困难，但是全市上下凝心聚力、共识共为，我们排难而进、奋起赶超有强大的合力。今年的政府工作，以科学发展观为指导，按照市委“两区互动、强工兴城、科学发展、奋起赶超“的决策部署，抓住关键，突破重点，全面落实，努力实现“三年财政翻番、五年全面进位“首战告捷。今年全市经济社会发展的主要预期目标是：生产总值增长13%，完成820亿元，力争突破840亿元；财政总收入增长15%，完成76.1亿元，力争突破80亿元；全社会固定资产投资增长30%以上，突破600亿元；社会消费品零售总额增长18%；居民消费价格总水平涨幅控制在4%以内；城镇居民人均可支配收入和农民人均纯收入增长10%；城镇化率提高1.6个百分点；城镇登记失业率控制在4.5%以内；万元生产总值能耗和化学需氧量、二氧化硫排放量控制在省政府下达的指标之内；人口自然增长率控制在8‰以内。为实现上述目标，主要抓好以下几个方面的工作：

（一）做强园区，推动工业经济跨上新台阶

工业大则总量大，工业强则实力强。在把握大局上突出工业这个核心，在资金投入上突出园区这个平台，在服务发展上突出项目这个重点，营造不遗余力建园区、倾心竭力上项目、举全市之力主攻工业的浓厚氛围，形成大手笔建大平台、大气魄上大项目、大作为成大产业的工业发展新格局。实现全年工业投资450亿元，增长50%，规模以上工业增加值300亿元以上，主营业务收入突破1000亿元。

创新园区建设，做实工业平台。把开发区、园区作为发展工业的主战场，创新体制，夯实基础，做优环境，提升人气，使之成为发展自主权最大、人员配备最强、办事效率最高、设施条件最优的工业平台。一是提升园区在发展中的地位。按照一级准政府的体制，给足园区充分的发展权、管理权，把发展重心定在园区，把精兵强将调往园区，把财力物力投向园区。对园区要提高职级、健全职能、给足职数，组成懂经济、善操作、有激情的园区工作团队。政府主要领导要坚持每月到园区现场办公，协调处理园区建设发展中的重大问题。各级部门要把园区作为履职尽责、服务发展的重点，改进方式，提高效能，凡是能移交的事权全部移交到园区办理，实行园区项目联审代办。二是推进重点园区建设。调集整合市直部门有效力量，推动九江开发区在“三区合一”的框架下高效协调运转，确保国家级开发区申报成功，支持城东工业集中区成长壮大，率先做大市本级。建立完善园区项目利益共享机制，促进园区加快同类企业、关联企业集聚，打造特色园区，谋求互惠发展。加紧盘整清理园区闲置土地和空置厂房，为新进项目落地和返乡农民工创业腾出空间。三是加大园区基础设施投入。将20%以上的城市土地经营收入用于园区建设，加快完善园区水、电、路、环保等配套设施。努力实现园区主营业务收入两年翻一番，力争三年内7个园区主营业务收入超百亿，其中九江开发区达到500亿元、财政收入突破10亿元。

推进重大项目，做强工业支柱。瞄准重大项目抓落户，围绕龙头企业搞扩改，改变工业“旗舰”不大、“航母”不多的现状，形成大项目带动、大企业支撑的良好势头。加强项目审核和调度，坚决防止以小报大、以弱充强、以假乱真；坚决防止抓而不实、推而不动、建而不快；认准大项目、抓住大项目、建成大项目。一是竣工投产一批，确保九钢150万吨板材、巨石玻纤、旭阳雷迪多晶硅、蓝星有机硅扩能、龙达化纤一期、诺贝尔陶瓷、赛得利二期、天赐日用化工、志高空调等项目年内投产。二是续建推进一批，加快建设亚泥四期、海天装备制造、城门山铜矿扩能、恒基伟业太阳能、安拿度环保陶瓷、金鼎钨钼矿业、锦兴纺织等项目。三是开工建设一批，力争江铜60万吨硫铁矿和40万吨铅锌冶炼、五洲电路板制造、鸭鸭创新基地、中粮30万吨植物油、维科印染退城进园、鑫旺矿业、九江县80万吨硫酸等项目年内开工。四是前期准备一批，积极跟进九江电厂四期、化工仓储物流等项目，围绕太阳能光伏、钢铁深加工、汽配船配、建材等优势产业，有针对性地包装、对接一批项目。强化激励机制，兑现工业上台阶奖和企业贡献奖（300万元）、项目引进奖（200万元），鼓励工业上台阶、企业作贡献、单位和个人引项目。

扶持中小企业，做大工业集群。针对金融危机给企业生产经营造成的冲击和影响，特别要帮助危机影响大、竞争实力弱的中小企业度过难关，从政策、资金、市场等方面加大扶持、强化服务。落实省政府“两降三促一优先”政策：降低园区新招工人社保门槛、降低劳动力培训成本，促进产业转移对接、促进劳动生产力提高、促进劳动就业，优先安排返乡农民工就业。落实出口退税和增值税转型政策，鼓励企业增加技改投入。落实成长型、就业型困难企业的税费缓交或减免政策。建立政银企风险共担机制，扩大担保融资，“以存促贷”融资，支持上市融资，开展股权融资，帮助企业增加现金流。严厉查处乱检查、乱收费、乱罚款、乱摊派等行为，切实减轻企业负担。帮助企业创品牌、争订单、拓市场。引导龙头企业引进、带动关联企业，促进中小企业融入龙头企业供应链，加快形成产业集群。

加快国企改制，做活工业存量。一是坚持全盘统筹。统一打包、统一政策，将所有待改制企业资产整合起来，在更大范围、更宽领域配置资源，归集改制资金，妥善安置职工，增强改制攻坚的整体实力。二是搞好市场运作。重视通过招商引资推进企业改制，多渠道、多途径吸引外资参与重组改造，促进企业重组重生，帮助职工就业创业。三是盘活企业资产。坚持改制一户企业，盘活一宗土地，培植一处财源，形成一个经济增长点。在办证、规费等方面给予优惠，在确权、交易等方面给予融通，积极推进德福电子、封缸酒厂、九针服装、喜敦啤酒等企业退城进园。四是强化资本运作。建立和完善国有资本经营管理制度，充分发挥国有资产经营公司债务重组、资金周转和发展投资等职能，加速市属国有资产经营运作，确保国有资产保值增值。

（二）振兴城区，推动城市建设走进新时代

发挥城市化对新型工业化和农业现代化的撬动作用，以拉大框架、完善功能、提升品位、繁荣经济为主线，规划坚持高品位、建设突出快速度、质量追求高标准，推动城建重心走进“八里湖时代”。

科学规划城市。按照2015年之前实现中心城区100平方公里、100万人口的目标，遵循主攻新城区、挺进八里湖、完善老城区、统筹庐山区、挖掘古浔阳、拉大中心城区框架的思路，年内完成城市总体规划修编，配套完善土地、水系、路网等各类专项规划，重点做好八里湖、甘棠湖、南门湖、十里河、龙开河水系规划，使之自然涵养、畅通活化、旱涝可调。按照“行政文化中心、最佳人居新区、休闲观光乐园”的定位，高起点、高品位编制八里湖组团详规。强化规划的刚性约束力，建立健全城乡规划监管机制。

科学建设城市。坚持城市建设项目化，把城市“西拓、东延、南移、中疏”落实到具体项目上。每年新增投入10-20亿元，突出建设八里湖组团，协调推进其它组团，逐步把九江建设成为一个山水辉映、环境优美、功能完善、宜居宜业的沿江港口城市。按照“六个一”的机制，责任到牵头领导、操作到具体人员、工期到时间节点，快速推进城市建设。竣工一批，确保长虹西大道在国庆节前竣工，完成十里河整治工程、环八里湖道路、八里湖滨湖文化生态公园、南山路、九莲南路、螺丝山路、第二垃圾处理场建设；续建一批，推进八里湖污水处理厂二期配套管网工程、龙开故道、胜利公园景观和绿化配套工程、环庐山公路交叉口改造；开工一批，建设南山公园、濂溪大道、桃园路、三马路南段；规划一批，启动八里湖市行政中心、国际会展中心的选址和设计。加强指导，强化考核，鼓励和促进各县（市、区）加快县城和中心集镇建设。

科学经营城市。加快城建靠资金，资金来源靠经营，经营重点在土地。十分珍惜土地资源，加大规费征缴力度，千方百计为做大城市筹集资金。一是加强土地运作。高度垄断土地一级市场，有序放开二级市场，规范交易行为。针对当前土地市场不景气的情况，加强城市土地盘整和清理，解决好违法违规用地、拖欠土地出让金、长期闲置不用等问题，依法收回、尽快收储，变“卖地筹资”为“储地融资”。坚持先规划后建设、先收储后出让、先造环境后卖地，成熟一块出让一块，做到净地出让，防止粗放供地，确保土地运作经济效益最大化。二是加强规费收缴。对城市出让土地和城建项目的所有规费，严格制度，严肃纪律，不开口子，不打折扣，坚决做到联审联批、一票总收、应收尽收。三是加强资产融资。在放大城市有形资产效益的同时，通过对城市无形资产使用权、经营权、冠名权等相关权益的市场运作，变无形资产为有效资本。充分发挥城投公司功能，积极探索运用金融产品缓解城建资金制约的新路子。

科学管理城市。管理见水平，管理出品位，品位显魅力。一是实施“创建工程”，开展文明城市、卫生城市、园林城市、国家级历史文化名城创建活动，以净化、美化、亮化为重点，加强“三区”结合部、城区与园区结合部、城乡结合部建设和管理；加强城市古建筑保护和修复，挖掘古浔阳文化内涵，对自然景观注入文化意蕴。二是实施“绿化工程”，全面推进中心城区扩地增绿、拆墙透绿、立体披绿，最大限度增加绿化总量，打造绿化精品，逐步形成一路一特色、一街一景致。三是实施“畅通工程”，疏通交通瓶颈，构建街区微循环，增加停车场所，缓解干道压力，强化交通管制，倡导文明出行。理顺体制，完善线网，更新车辆，提升公交档次。四是实施“平安工程”，加强城市环境保护和综合治理，保障城市饮水、生态和人居安全。构建数字化城市管理平台，完善城市公共服务，加强应急救援队伍建设，增强城市应急保障和防灾减灾能力。

科学繁荣城市。振兴城市，重在兴业；繁荣城市，重在惠民。城市不是楼宇和街道的简单交织，而是经济和文化的集优配置，更是人们宜业宜居的美好家园。让更多的人分享城市发展成果、享受现代城市生活，是政府不懈的追求。繁荣城市经济，一是提升传统服务业，完善商业网点布局，加快形成一批专业特色街、品牌旗舰店和大型卖场，打造中心商贸区；支持京九农副产品批发市场改造升级，推进华东装饰材料市场、南方粮食交易市场、九江国际汽车城等重点市场加快发展。二是发展现代服务业，扩大连锁、代理、配送等现代营销，搞活总部经济、楼宇经济、社区服务、保健医疗、职业培训、文化娱乐、服务外包、健身休闲，规范会计审计、公证评估、监理咨询、鉴定拍卖、法律服务。三是做大城市物流业，加快港口和电子物流信息平台建设，发展水陆联运，支持九江机场扩大空运，积极开辟物流新通道。四是改善城市住房条件，合理确定经济适用房、廉租房和商品房比例，正确引导房价和市场预期，促进房地产市场健康发展。

（三）夯实基础，推动“三农”工作迈出新步伐

“三农”问题的核心是农民，农民问题的关键是增收。坚持“三农”基础地位不动摇，应始终致力农民增收，始终致力农业增效，始终致力农村改善，把农村的各项事业办好。

推进农村制度改革。实行最严格的耕地保护和节约用地制度；允许土地承包经营权依法、自愿、有序流转，推动适度规模经营；深化林权制度配套改革，规范林业产权交易，拓展林权质押贷款和林业保险业务；推进国有农场税费改革和综合配套改革；落实强农惠农政策，将种粮、良种、生猪养殖、农机具购置和农用成品油、化肥价格补贴等发放到位；完善补偿和保障机制，确保失地农民生活水平有提高，长远生计有保障。

强化农村发展基础。实施“沃土工程”、“造地增粮富民工程”、“新增百亿斤优质商品粮生产能力工程”，改造中低产田8万亩。全面打胜“公路建设翻身仗”，新建农村水泥路1500公里，砂石路280公里，实现100%行政村通公路，力争95%的行政村通水泥路；新开工改渡建桥35座。推进鄱阳湖二期防洪工程；加快推进一批农田灌溉、小农水、水利血防、农发水保等项目；积极倡导和鼓励社会办水利。切实抓好新增1134个新农村试点村建设，以 “三清七改五普及”和“三绿一处理” 为切入点，加快推进村庄整治；重视产业培育，力争80%的建设点形成特色产业；抓好45个大中型水库移民新村建设；开展“文明和谐村庄”创建活动，培育新型农民，促进乡风文明。

壮大农业产业规模。高度重视粮食安全，粮食种植面积稳定在360万亩以上，总产量27亿斤以上。大力培植优势产业，发展特种水产和水产品加工，抓好标准化农产品生产示范基地和鄱阳湖现代农业产业带建设，加强鄱阳湖渔业资源深度开发，巩固发展九江水梨。推进农业项目建设，每个县(市、区)力争引进3000万元以上农业项目2-3个，亿元以上项目1-2个，市直农口各单位引进3000万元以上农业项目1-2个；积极申报一批农村经济社会发展项目，力争更多地争取国家和省级农业项目资金。培育壮大龙头企业，重点培植3个年销售收入超10亿元、5个超5亿元、20个超亿元的龙头企业，新增优质农产品基地50个；开展“一乡一业”、“一村一品”建设，壮大特色种养业，力争新增绿色、有机、无公害食品品牌30个，新增国家级、省级名牌农产品和著名、驰名商标10个以上。

完善农业服务体系。加大农业科技投入，抓好“粮食丰产科技工程”等农业科技计划项目的实施。加强农业生产资料调配和质量监管，逐步完善基层农业技术推广、动植物疫病防控等公共服务体系，完成市级畜牧兽医服务体系建设，启动市级农产品质量安全检验检测中心建设。鼓励农民专业合作社发展，壮大农村经纪人队伍，着力破解大宗农产品“卖难”问题。

（四）扩大开放，推动项目投资取得新突破

坚持开放引领，突出投资拉动，以开放引项目，以项目扩投入，以投入保增长。

加大招商引资力度上项目。抓住全球投资看好中国、国内客商看好中部的有利时机，创新开放举措，加快产业和市场对接，针对性地组织招商活动，努力提高招商实效。一是健全机制促招商。充分发挥政府的主导作用和企业的主体作用，营造全社会重视招商、全市上下合力抓招商的良好氛围。充实力量，强化责任，加强激励，形成主动、灵活、务实的招商机制，做到大项目有领导牵头、客商有专人跟踪、任务有项目支撑，年终考核，奖惩兑现。二是突出重点抓招商。坚持优势资源招优势企业，战略资源招战略投资者。紧紧盯住重点地区、重点产业、重点企业、重点客商，瞄准全球500强和国内200强企业敲门招商，瞄准中央、省属国有大型企业定向招商，瞄准与我市产业关联度高、带动能力强的行业龙头企业开展产业招商，力争全市实际利用外资4.8亿美元，增长10％；实际引进省外5000万元以上工业项目资金135亿元，增长20％；外贸出口总额3.2亿美元，增长20％。

加大对接跟踪力度争项目。用足用好国家扩大投资、拉动内需的各项政策，研究动向，超前论证，科学包装，频密对接，全程跟踪，力争全市争资份额超过全省平均水平。加快在建项目进度，竣工完成316国道改造、星子大岭风电、沙城和永修220千伏输变电工程等项目。续建推进昌九城际铁路、京九铁路和武九铁路电气化改造、杭瑞高速九瑞段、彭湖高速公路、九江至瑞昌码头沿江快速通道、105国道改造、湖口500千伏变电站等项目。开工建设彭泽核电场地平整工程、九景衢铁路、九江长江二桥、庐山西海高速公路、214码头改扩建、中石油天然气管道工程、省天然气输气母站等项目。切实加强与上级部门的沟通和对接，积极做好彭泽核电主体工程、九江绕城高速、老爷庙风电、长岭风电二期、武九客运专线、永修火电、瑞昌火电、九江至长沙铁路、九江至池州城际铁路、都昌蔡岭至九江县刘家畈加密高速、大广-京珠高速连接线等项目前期工作。

加大环境整治力度落项目。树立环境也是生产力、改善环境就是发展生产力的理念，把创优项目落户环境作为推进项目建设的生命线。积极打造透明的政策环境、快捷的办事环境、诚信的市场环境、公正的法治环境。强化项目保障措施，加强项目调度，及时解决重大项目推进过程中的各类困难和问题；推行重大项目涉政事务代理制，帮助企业办理项目论证、环评安评、土地报批，畅通重大项目建设绿色通道。痛下决心，重拳出击，着力整治项目落户速度慢、项目推进阻力多、外商办事麻烦多、企业经营干扰多的“中梗阻”顽症，严厉查处一批破坏投资环境的人和事，清除害群之马，营造安商、亲商的发展氛围。

（五）激发活力，推动县域经济实现新跨越

按照三年实现财政收入县县过5亿，一批县区过10亿、15亿的目标，放开放活，强化激励，八仙过海，各显神通，促进县域经济做大做强。

给足发展权。理顺市县行政事权和税收体制，全面落实“省直管县”财政政策。在立项、规划、用地、环评、安评等经济管理权限上，可以下放到县区的全部下放，使其在发展经济上有更大的自主权。全力以赴帮助县区争取政策和资金支持，凡是县区享受的，市里一律不截留；全力以赴加大对县区项目的帮扶力度，凡是纳入市里调度的项目，跟踪协调到底；全力以赴帮助县区解决发展难题，加快完成第二轮土地规划修编，凡是涉及县区经济发展的问题，市里及时跟进，快速解决。

铺好就业路。农民工是工业化、城市化的参与者和建设者，是活跃县域经济的生力军。受金融危机影响，农民工就业遇到了暂时困难，我们要以高度的社会责任感做好返乡农民工的就业创业工作。在有序组织劳务输出的同时，鼓励和支持农村富余劳动力进城镇、进园区，从事生产制造、建筑施工、社区服务，实现就近就地就业；免费为工业园区新招员工进行技能培训，完善人力资源供求平台，强化培训机构与企业、缺工企业与大中专毕业生和农民工的岗位对接。搭建园区招工培训平台，搞好职业技能培训，为农村劳动力转移就业提供服务。

掀起创业潮。按照“非禁即可”的原则，放宽准入领域和登记条件。对初创小企业可实行“零资金”注册，降低创业成本；落实扶持政策，设立各种形式的扶持基金，减免或缓交有关税费，鼓励返乡农民工、下岗失业人员、退役士兵、大中专毕业生和归国留学生创办加工型、服务型、科技型企业，促进个体工商户转办私营企业，支持中小企业“二次创业”；加大金融支持，发挥各类担保公司作用，对符合条件的工业和商贸流通企业实行“零担保费”担保。引导金融机构探索开展股权质押、林权抵押等贷款业务。为返乡农民工办理创业贷款“惠农卡”，帮助解决融资难题；大力宣传创业先进典型，营造浓厚的舆论氛围，掀起新一轮全民创业热潮。

打好擂台赛。进一步完善县域经济考评体系，强化激励措施，每年设立500万元奖励资金，对县域经济考评位列全市前三名的县（市、区）及其党政主要负责人进行奖励。按照“保基数、分增量”和“共同投入、利益共享”原则，市、县两级根据投入比例和实际贡献划分收益。

（六）扬优成势，推动特色板块形成新亮点

潜在优势是奋起赶超的有利条件，扬优发展是奋起赶超的现实选择，必须深挖潜力，放大优势，做强特色。

扬沿江水运之优，成临港产业集聚之势。强化产业支撑，加大投资强度，完善基础设施，推动沿江开发向纵深发展、向腹地延伸。坚持把引进工业项目、做强沿江产业，作为沿江开发的生命线。加快引进一批重化工业项目，促进钢铁、造船、建材、化工等临港特色产业集聚。力争城西板块引进亿元工业项目10个以上，其中10亿元以上项目2个；其它板块各引进亿元工业项目2个以上。编制完成城西港区二期控规，完善路网、码头、通关中心、物流园区、安置小区等配套设施，推动电子口岸建设，争取九江口岸扩大开放和纳入第七类废料进口转关口岸；加快壮大城东工业集中区，启动建设赤湖二期和银砂湾工业区，协调推进码头、马土当板块，提升沿江工业平台承载能力。加强投融资平台建设，力争城西港区融资5-10亿元，其它板块各融资1-5亿元。

扬庐山品牌之优，成全市旅游联动之势。加快理顺旅游管理体制，加强旅游资源整合，努力形成以庐山为龙头，山上山下互通、景区景点互动、文化旅游互融的大旅游格局。制定完善市县旅游发展总体规划和重点旅游景区控制性详规。加快推进庐山西海国际艺术园、东林大佛景区、庐山园门服务中心、威家旅游商贸中心等重大旅游项目建设，启动一批景区停车场、游步道、星级酒店等配套设施建设，提升旅游接待服务水平。加强区域协作，设立促销基金，进一步完善经营机制。加大旅游产业融资力度，广泛吸纳外资、民资进入，激发旅游经济发展活力。开展丰富多彩的文化旅游活动，策划创作彰显九江地域特色的大型文化演绎剧目和影视精品，提高魅力九江的知名度和美誉度。

扬湖区生态之优，成绿色经济衍生之势。突出生态保护和产业发展，积极融入鄱阳湖生态经济区建设。超前包装和推进一批湿地保护、水土保持、生态补偿、生态移民、血吸虫病防治、湖区中心码头等项目，不断改善湖区生产生活条件；着力引进一批新型制造、优质农产品基地、生态旅游和清洁能源项目，加快湖区经济发展；规划建设一批沿湖生态县城和村镇，提高湖区城镇化水平。推进重点领域和重点行业节能降耗，实施永修云山经济开发区国家级循环经济试点；加快污水处理设施建设，力争全市所有县（市）污水处理厂7月1日前投入运行；推进农村生活垃圾无害化处理；抓好共青城、德安县和都昌县沿湖水质监测站建设；加强修河源头保护。

扬共青独特之优，成青年创业蓬勃之势。紧紧抓住省委、省政府和团中央高度关注、高位推动共青城发展的机遇，认真贯彻落实好各项帮扶措施，推进共青城加快发展。加快完成共青城各类规划编制；从争资争项、用地指标、项目报批上给予倾斜，加快推进南湖大桥、工业大道、输变电站、国际青年论坛永久会址、鄱阳湖物理模型等重点工程建设；突出抓好青年创业基地建设，加紧引进一批新型产业项目，做大做强产业支柱；争取共青城享受县区同等待遇，激发体制机制活力，努力把共青城建设成为在国内外享有盛誉的现代化的社会主义开发区。

（七）改善民生，推动社会事业再上新水平

按照人民政府为人民的要求，结合九江实际，尽最大的努力实施民生工程，尽最大的努力办好惠民实事。重点围绕八个方面，办好89件实事。

一是努力扩大就业。着力办好8件实事，主要是：以创业带就业，新增小额担保贷款1.87亿元，支持个人创业和劳动密集型小企业发展。加强就业培训，培训6.6万农村劳动力；免费为5.1万下岗失业人员、城镇登记失业人员、未就业高校毕业生、城镇复员退伍军人提供技能和创业培训。扶持困难群体就业，确保有就业愿望和能力的零就业家庭至少有1人就业，为残疾人购买390个公益性岗位。将符合条件的被征地农民、农林水失业人员、未安置的城镇退役军人等统一纳入失业登记范围，享受就业扶持政策。力争新增城镇就业5.21万人，其中 “4050”人员5400人；新增转移农村劳动力4.3万人，其中省内转移2.41万人。

二是进一步提高社会救助能力。着力办好14件实事，主要是：提高城乡低保对象补助标准，月人均补差分别达到155元、65元。提高农村五保户供养标准，年人均提高60元。提高上世纪六十年代精简下放老职工救济金水平，城乡月人均分别达到200元、160元。提高城镇“三无”特困群众供养标准，月人均提高到300元。提高义务兵优待金标准和退役士兵自谋职业补助水平，增加企业军转干部和部分退役军人生活补助。完善临时救助制度，配套资金156万元，对因突发性事件造成生活困难的群众实行临时救助。建立因自然灾害死亡人员抚慰金制度，按每人5000元标准抚慰家属。对困难群众实施法律援助，完成法律援助案件1200件以上。对符合条件的计划生育扶助对象，每年发放720元奖励扶持金，让计划生育家庭真正得到更多实惠。新建市儿童福利院和2所县级综合福利院。为1万名残疾人提供康复服务，为900名贫困白内障患者实施复明手术，为2400名贫困精神病患者实施医疗救助，为100名贫困残疾人配发辅助器具，让残疾人感受到更多的关爱和温暖。

三是不断提升社会保障水平。着力办好9件实事，主要是：扩大养老保险覆盖面，城镇职工参保人数达61.99万人。提高企业退休人员养老金水平，人均增加140元。推进被征地农民养老保险试点，新增参保1.5万人。扩大城镇职工工伤保险覆盖范围，参保人数达25.35万人。提高失业保险统筹水平，参保人数达34万人。调整园区职工社保缴费基数和计算标准，接续返乡农民工社会保险关系。改扩建40所农村敬老院，建设一批县区老年公寓。对离任老村支书、村主任给予生活补贴，对敬老院、福利院、光荣院“三院”中符合条件的工作人员以及居委会工作人员给予工作补贴，让长期工作在最基层的同志生活上得到应有照顾。

四是大力加强医疗保障和食品药品安全。着力办好15件实事，主要是：扩大城镇职工基本医疗保险覆盖范围，参保职工达51.2万人。完善城镇居民基本医疗保险制度，将大学生全部纳入城镇居民基本医疗保险范围。提高新型农村合作医疗保障水平，参合农民达到315万人，统筹基金使用率达85%。全市配套救助资金1477万元，提高大病医疗救助水平。免费向符合条件的城市居民发放公共卫生服务卡490万元。完善城乡卫生服务体系，建立100家农村示范药店，推进344个村卫生室（所）、72个社区卫生服务机构、13所乡镇中心卫生院、2所县级综合医院、1所县级中医院达到建设标准。新招聘农村卫生专业技术人员1000名，免费培训农村卫生技术人员2000名。按每人每年1000元标准对符合条件的村卫生室执业医生给予补助。乡镇卫生院工作人员基本工资全部由差额转为全额保障。

五是继续大力推行济困助学。着力办好10件实事，主要是：增加城乡义务教育发展经费，继续实施“两免一补三资助”。为义务教育阶段59.5万名学生免除学杂费、免费提供教科书，对3.67万名义务教育阶段家庭经济困难学生补助寄宿生活费，资助家庭经济困难的高中生1万人以上、中专生2.8万人以上、大学新生2700人以上。免费录取540名家庭经济困难学生入读技工学校。新招聘1000名农村教师。改造农村中小学校舍8万平方米。对边远地区教师发放特殊津贴。今年所有县（市、区）公务员津贴补贴和义务教育教师绩效工资要全部发放到位。

六是加大保障性住房建设力度。着力办好5件实事，主要是：扩大廉租住房保障，力争新建廉租住房5000套，租赁补贴1.2万户。继续推进经济适用住房建设，受益家庭达到1570户。启动实施中心城区20万平方米棚户区（城市低洼危旧房住宅区）改造。建立农村困难群众危房救助制度，支持帮助732户农村困难群众改造危旧住房，逐步改善困难群众的住房条件。

七是扎实推进扶贫开发。着力办好3件实事，主要是：继续实施移民搬迁扶贫，力争完成深山区、库区、地质灾害频发区贫困群众5600人移民搬迁。大力实施“整村推进计划”，重点抓好357个重点村扶贫开发。推进农业产业化扶贫，扶持产业化扶贫龙头企业发展和产业化基地建设，扩大贫困村互助资金试点范围，帮助贫困农户通过发展生产增加收入、改善生活。

八是切实改善人民群众生产生活条件。着力办好25件实事，主要是：改善群众出行条件，开通城区至城西港区等郊区公交线路。加大改水力度，新增解决6万农村群众安全饮水问题。推行清洁能源，增加农村沼气用户2.28万户。加强农村环卫设施建设，新建农村家庭生活垃圾处理场692个。实施“万村千乡市场工程”，新建标准化农家店100家以上。实施农村光缆进村工程，使行政村宽带网络通达率达到90％以上。实施广播电视村村通工程，帮助654个20户以上自然村开通卫星电视。建设乡镇文化站39个，建成农家书屋219个。开展农民体育健身场所工程建设试点，新建农民体育健身场所51个。进一步优化城区人居环境，完成城区自来水一户一表改造1.5万户以上，启动江州菜市场等一批农贸市场升级改造。进一步完善公共服务体系，设立就业服务热线和网络平台，整合阳光政务热线，畅通民声通道。在端午节、儿童节、中秋节、重阳节，向全市人民免费开放观光游览景区景点。

与此同时，全面发展社会事业。一是优先发展教育。推进义务教育均衡发展，进一步解决农村留守儿童和进城务工农民子女接受义务教育问题；办好特殊教育，切实改善残疾少年儿童的教育条件；加快普及高中教育，积极推进普通高中教育新课程实验；大力发展职业教育，强化与就业的对接，切实提高就业率；做强做优高等教育，不断提升高校办学水平。二是推动科技进步。继续开展创建全国科技进步县(市)活动，加强科技知识普及等各项工作；巩固科技-人才-项目对接成果，加快引进高新技术项目，加快科技成果转化，力争科技进步对经济增长的贡献率提高5个百分点。三是加强卫生建设。推进医疗卫生体制改革，增强医疗卫生机构的动力和活力；强化传染病疫情网络直报管理，提高重大传染性疾病防控和应对突发公共卫生事件的处置能力；广泛深入开展城乡爱国卫生运动，抓好妇幼卫生和健康教育工作；积极发展中医药事业。四是大力发展文体事业。推进公共文化服务体系建设，支持广电、报业、出版等文化产业加快发展；加强优秀传统文化的挖掘和利用，重视历史文物、文化古迹、革命旧址遗址和非物质文化遗产的保护。广泛深入开展全民健身运动，实施农民体育健身工程，提高竞技体育水平。五是做好人口人才工作。健全计划生育利益导向机制，稳定低生育水平，有效治理出生人口性别比偏高问题，提高出生人口素质。加大高层次人才引进力度，为各类人才营造引得进、留得住、用得好的干事创业环境。进一步加强国防建设，做好征兵和民兵预备役工作，启动新一轮“双拥创建”活动，改善人防设施。强化统计、审计、档案、编制、信息化工作，提高经济社会管理水平。加强对台、外事侨务和民族宗教工作，促进交流，增进团结。发挥群团作用，协调发展工会、共青团、妇女儿童、老龄事业，支持发展红十字人道和慈善事业，推动社区志愿服务和救护培训工作；充分发挥地震、气象、水文等工作在防灾减灾中的作用。

三、加强政府自身建设

2009年将是我国改革开放以来，尤其是新世纪以来经济最困难、形势最复杂的一年，我们既要有充分的估计，又要有坚定的信心。困难面前勇者胜。我们一定要把思想行动统一到胡锦涛总书记视察江西时提出的“三保一弘扬”要求上来，统一到省委省政府准确分析新形势、积极应对新挑战所作出的一系列重大部署上来，统一到市委确定的目标任务和战略抉择上来，以“对党负责、为民服务、高效务实、清正廉洁”的政府团队精神，全力以赴，攻坚克难，把强工兴城的决策落实好，把奋起赶超的目标实现好。

（一）提高操作能力保增长。增长的速度决定着赶超进位的跨度。操作决定成败，操作的核心在于化解危机、破解难题，提高效能、落实到位。一是明确责任抓操作。坚定地按照市委九届七次全会确定的任务，变目标为责任，化责任为行动；明确地紧盯省政府“6+1”考评体系，强化考核激励，加强跟踪督查，实行分管负责、丢分问责，形成分兵把口、责任到人、落实到点、工作到位的干事成事机制。二是突出时效抓操作。奋力赶超必须增强速度意识，强化时间观念。赶超在于速度，速度不快就会继续后退；时间就是金钱，拖长时间就会增加成本。要瞄准既定目标加速度，掐住时间节点抢进度，快节奏推进项目，超常规破解难题，夜以继日，争分夺秒，一切工作往前赶。三是合法高效抓操作。方法是成功的基础、理由是失败的借口。操作到位在于方法创新、融通办事，只为合法高效想办法、不为推诿扯皮找理由。深入开展“机关效能年”活动，按照“三个减少30%”的要求，精简审批项目、减少审批环节、缩短审批时间、加强审批监督。对重大产业项目，推行联合审批、现场审批、特事特批，努力使我市行政审批在全省时间最短、效率最高。四是强化服务抓操作。切实增强服务观念，提高服务能力，在政策服务中突出灵活操作，在资金服务中加强融资操作，在项目服务中搞好融通操作，在服务群众时注重有情操作。

（二）提高统筹能力保民生。政府最大的追求就是事业的成功，政府最好的口碑就是人民的满意。一手抓发展，一手托民生，要领在于统筹，统筹的关键是突出中心、突破重点，顾全大局、兼顾全面。一是在统筹中走活一盘棋。维护核心党委讲政治，围绕经济中心干实事。以“两区互动、强工兴城、科学发展、奋起赶超”为重点，围绕经济建设中心，上下一个调，拧成一股绳，凝神聚力抓发展，同心同德谋赶超。二是在统筹中解百姓之忧。统筹的重点是帮困者，扶弱者，要点是把实事办到群众的心坎上，把好事办到群众的急需处。急群众之所急，帮群众之所需，办群众之所盼，切实提高政府的公信力和人民群众的满意度。三是在统筹中践行科学发展观。在“强工兴城”的同时，夯实“三农”基础，协调发展文化旅游和现代服务业；既注重鞭打快牛，促进基础强、条件好的地方加快崛起，也注重均衡推进，支持基础弱、条件差的地方奋力赶超。在鼓励能人创业的同时，关注群众就业和弱势群体生活。在加快发展的同时，注重建养生态，不以牺牲环境换取一时增长，促进人与自然和谐发展。

（三）提高驾驭能力保稳定。今年，政治敏感阶段相对集中、经济社会矛盾叠加交织、影响社会稳定因素增多，保稳定的任务重、压力大，要进一步增强责任感，提高驾驭复杂局面能力。一是提高预见力。全面掌握社会动态，敏锐洞察矛盾，对社会各方面的诉求及时回应，争取主动权；对苗头性、倾向性的问题，早发现、早调处，力争化解于萌芽状态。二是提高掌控力。增强安全发展意识，落实维护安全稳定责任制。加强安全整治，坚决遏制重特大安全事故发生；完善利益协调、诉求表达、矛盾调处机制，畅通信访渠道，规范信访秩序，实行领导包案、开门接访，完善矛盾问题研判、会商机制。三是提高处置力。敢于正视矛盾，善于化解矛盾，妥善解决社会和群众关注的热点难点问题；以“平安创建”为抓手，健全社会治安防控体系，加强社会治安综合治理，严厉打击各种犯罪，重拳开展“打黑、除恶、治霸”行动；进一步健全应急工作机制，动员运用社会各方面资源，提高突发性事件处置能力和社会治安局势的控制力，确保社会治安大局稳定。

（四）提高拒腐能力保清廉。坚持用法律和纪律规范执政行为，不断加强政府民主法制建设。一是依法行政。自觉接受市人大和市政协的监督，认真办理代表建议、议案和委员提案，广泛听取各方面意见，接受社会公众监督；积极开展法治城市、法治县（市、区）创建工作，推进普法工作和依法治理；深入推进政务、村务公开，规范和公开行政处罚自由裁量权，完善信息公开发布机制，推进县（市、区）公共资源交易中心建设；进一步理顺部门职责，推进市县行政机构改革。二是廉洁从政。认真贯彻落实党风廉政责任制和廉洁自律的各项规定，抓好政府各部门党风廉政建设和反腐败各项工作。把加强项目资金管理作为今年廉政建设重点，在各类投资项目建设中，制度约束先行，管理监督同步，检查审计跟进，坚决防止截留挪用、滞留不用、挤占乱用等现象，确保资金安全运行，确保项目建设质量。加强财政转移支付、土地出让、政府采购、产权交易的监管，严格查处违规违纪案件，规范行政权力运行，不给党的形象抹黑，不为自己留骂名。三是勤俭理政。千方百计挤出资金保重点保民生，树立过紧日子的思想，勤俭办一切事业，反对铺张浪费和奢靡享乐，做到公务用车、会议经费、公务接待费、出国（境）经费等支出“零增长”。

各位代表！三年翻番，只争朝夕；五年进位，时不我待。让我们在省委、省政府和市委的正确领导下，在市人大、市政协的监督支持下，坚定信心，应对挑战，齐心协力，奋起赶超，全面完成市委确定的各项目标任务,以优异的成绩向新中国60华诞献礼！
